# Enhancing spectral analysis in nonlinear dynamics with pseudoeigenfunctions from continuous spectra

**DOI:** 10.1038/s41598-024-69837-y

**Published:** 2024-08-20

**Authors:** Itsushi Sakata, Yoshinobu Kawahara

**Affiliations:** 1 RIKEN Center for Advanced Intelligence Project, Tokyo, Japan; 2 Graduate School of Information Science and Technology, Osaka University, Osaka, Japan

**Keywords:** Information technology, Applied mathematics, Nonlinear phenomena

## Abstract

The analysis of complex behavior in empirical data poses significant challenges in various scientific and engineering disciplines. Dynamic Mode Decomposition (DMD) is a widely used method to reveal the spectral features of nonlinear dynamical systems without prior knowledge. However, because of its infinite dimensions, analyzing the continuous spectrum resulting from chaos and noise is problematic. We propose a clustering-based method to analyze dynamics represented by pseudoeigenfunctions associated with continuous spectra. This paper describes data-driven algorithms for comparing pseudoeigenfunctions using subspaces. We used the recently proposed Residual Dynamic Mode Decomposition (ResDMD) to approximate spectral properties from the data. To validate the effectiveness of our method, we analyzed 1D signal data affected by thermal noise and 2D-time series of coupled chaotic systems exhibiting generalized synchronization. The results reveal dynamic patterns previously obscured by conventional DMD analyses and provide insights into coupled chaos’s complexities.

## Introduction

Spectral analysis breaks down complex data into simpler components, which is essential to understand the dynamics of various systems. Traditional methods like discrete Fourier transform and proper orthogonal decomposition (POD) have been widely used for mode decomposition^1,2^. Dynamic Mode Decomposition (DMD) is a more recent technique that isolates modes to provide insights into nonlinear systems, such as fluid dynamics, neuroscience, and quantum optics^3–7^. However, analyzing the continuous spectrum from chaotic and noisy data using DMD remains challenging.

Our study addresses this by proposing a clustering-based method using pseudoeigenfunctions derived from continuous spectra. This method involves grouping similar pseudoeigenfunctions to capture the dynamic structures within the data. By doing so, we aim to provide a more detailed understanding of the underlying dynamics, especially in systems influenced by noise and chaos. The detailed description of our clustering approach is provided in the “Methods” section, but this brief overview highlights its purpose and significance in our analysis.

The primary goal of DMD is to approximate the Koopman operator. This infinite-dimensional linear operator plays a central role in the analysis of nonlinear dynamical systems such as turbulence and molecular dynamics^8,9^. The Koopman operator provides a comprehensive framework for understanding the fundamental properties of these systems. To illustrate spectral analysis using the Koopman operator, consider a dynamical system with a state $\varvec{x}$ that follows an unknown nonlinear function $\varvec{F}: \Omega \rightarrow \Omega$ in a discrete-time state space $\Omega \subset \mathbb {C}^d$,

1 $$\begin{aligned} \varvec{x}_{n+1} = \varvec{F}(\varvec{x}_n), \; n \ge 0. \end{aligned}$$

The Koopman operator $\mathcal {K}$ is an infinite dimensional linear operator acting on observable functions $g:\Omega \rightarrow \mathbb {C}$,

2 $$\begin{aligned}{}[\mathcal {K} g] (\varvec{x}) = (g \circ \varvec{F}) (\varvec{x}), \; \varvec{x} \in \Omega . \end{aligned}$$

This operator can be decomposed into eigenvalues and eigenvectors:

3 $$\begin{aligned} \mathcal {K} \varphi _j (\varvec{x}) = \lambda _j \varphi _j (\varvec{x}), \end{aligned}$$

where $\lambda$ and $\varphi$ represent the eigenvalue and eigenvector, respectively. The observed signal $g(\varvec{x}_n)$ is decomposed into simpler parts with corresponding spectral frequencies:

4 $$\begin{aligned} g(\varvec{x}_n) = [\mathcal {K}^n g] (\varvec{x}_0) = \sum _{j=1}^{\infty } c_j \lambda _j^n \varphi _{j} (\varvec{x}_0), \end{aligned}$$

where $c_j \in \mathbb {C}$ is the $j$th coefficient. Since the Koopman operator is an infinite-dimensional linear operator, it can have a continuous spectrum. Since turbulence, chaos, transients, and noise contain continuous components in frequency^10^, the treatment of continuous spectra becomes inevitable in Koopman spectral analysis^11^. However, continuous spectra must be handled carefully because their presence can be obscured when reduced to finite dimensions for estimation purposes.

Residual Dynamic Mode Decomposition (ResDMD) provides a data-driven strategy for managing continuous spectra through spectral measures^12,13^. Importantly, ResDMD not only addresses the challenge of continuous spectra but also deals with issues like spurious modes that can arise in general systems. Spectral pollution, where discretizations cause spurious eigenvalues unrelated to the operator, is a well-known difficulty in computing spectra of infinite-dimensional operators^14–16^. Methods such as Extended Dynamic Mode Decomposition (EDMD) can suffer from this problem^17^. ResDMD offers a principled way to detect and avoid spectral pollution, allowing the computation of spectra of general Koopman operators without these artifacts.

Within the measure-preserving system, a set of eigenfunctions and parameterized generalized eigenfunctions on the unit disk are used to capture a representation of the mode that includes the continuous spectrum:

5 $$\begin{aligned} g(\varvec{x}_n) = [\mathcal {K}^n g] (\varvec{x}_0) = \sum _{\lambda \in \sigma _p (\mathcal {K})} {c}_{\lambda } \lambda ^n \varphi _{\lambda } (\varvec{x}_0) + \int \limits _{-\pi }^{\pi } e^{i n \theta } \phi _{\theta , g} (\varvec{x}_0) \text {d}\theta , \end{aligned}$$

where $\sigma _p (\mathcal {K})$ represents the set of eigenvalues of $\mathcal {K}$, and $\phi _{\theta , g}$ is a generalized eigenfunction. The first term in Eq. (5) corresponds to a discrete spectrum as described in Eq. (4), and the second term corresponds to a continuous spectrum. These decompositions can often characterize dynamical systems. ResDMD approximates the continuous aspects of this equation as a collection of discrete values, focusing on smoothing calculations using the resolvent operator^18,19^. ResDMD approximates the spectral properties described by generalized eigenfunctions through pseudoeigenfunctions. This method allows the computation of the continuous spectrum within the framework of the Koopman operator, marking a significant advance in the field.

We propose a clustering-based method designed to analyze the dynamics represented by pseudoeigenfunctions. Our approach aims to analyze and characterize the dynamic structures inherent in systems with continuous spectra. This research seeks to uncover dynamic structures previously inaccessible with standard DMD techniques, offering a new perspective on the intricate behavior of nonlinear dynamical systems.

To provide a brief overview, we construct a similarity matrix from the pseudoeigenfunctions derived through ResDMD using the kernel principal angle^20^. Using spectral clustering^21^, which is robust to noise and nonlinear structures while preserving similar structures, we classify the dynamics based on the continuous spectra revealed by the pseudoeigenfunctions. This method enables the identification of distinct dynamic behaviors and enhances our understanding of complex systems.

We demonstrate the effectiveness of our method by analyzing one-dimensional signal data affected by thermal noise and two-dimensional time series of coupled chaotic systems. In “Results” section, we present the results of applying our proposed method to noisy synthetic signals and coupled chaos. In “Discussion” section, we discuss the effectiveness of our method based on these results. In “Methods” section, we describe our proposed method for clustering pseudoeigenfunctions that reflect the characteristics of the dynamics.

## Results

To validate the effectiveness of our proposed method, we analyzed data that exhibit aperiodicity with continuous spectra. Specifically, we examined a one-dimensional synthetic signal influenced by thermal noise and computational simulation data from coupled chaotic systems.

**Figure 1:**
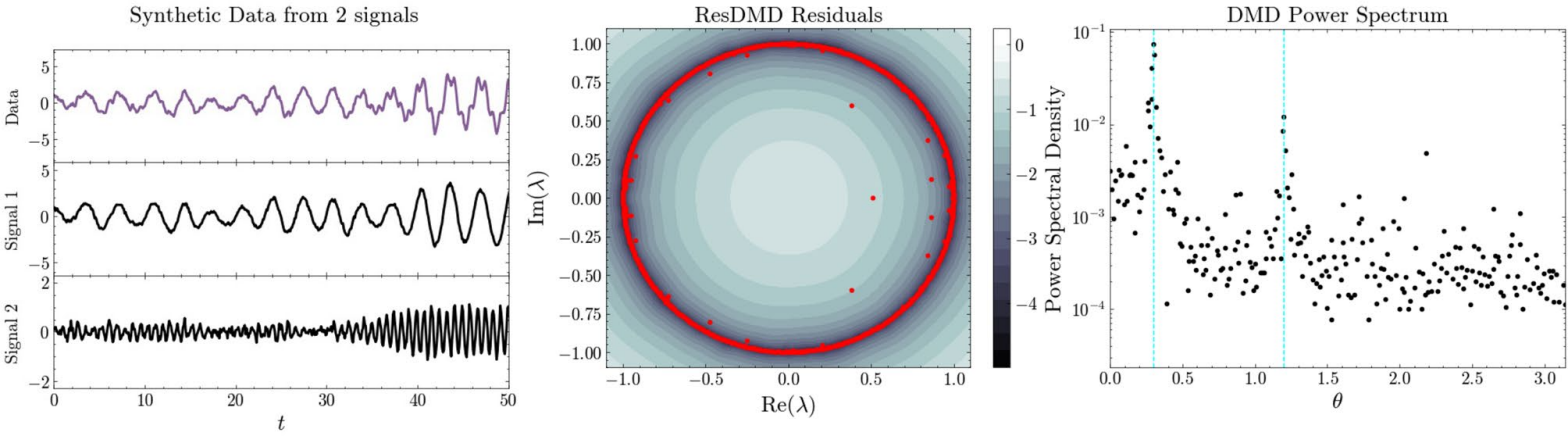
Left: Synthetic signal with aperiodicity; the data is an observed signal synthesized from signal 1 and signal 2 ($x_1 (t)$ and $x_2 (t)$ in Eq. (6)), which are harmonic oscillations with different frequencies and thermal noise. Middle: Pseudospectra (contours) and DMD eigenvalues (red dots) computed from the synthetic signal. The darker color indicates the common logarithm of the residuals computed by ResDMD. The darker the color, the smaller the residuals, indicating a distribution of DMD eigenvalues. Some spurious eigenvalues have large residuals. Right: Spectrum of DMD. The horizontal axis shows the argument of the complex ($\lambda = e^{i \theta }$) eigenvalues and the vertical axis shows the absolute values of the DMD coefficients. The two frequencies $\omega _1$ and $\omega _2$ in Eq. (6) are depicted as cyan vertical lines.

**Figure 2:**
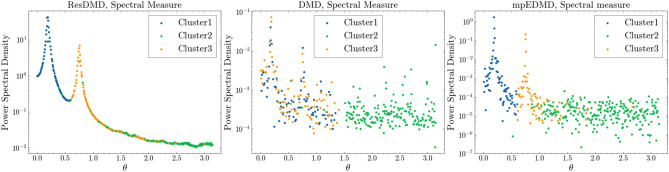
Left: Computed spectral measures of continuous spectra by ResDMD from the data shown in the left panel of Fig. 1. Middle: DMD eigenvalue spectra from the data shown in the left panel of Fig. 1. The colors (blue, green, and orange) indicate the individual clusters. Right: mpEDMD spectral measure.

### 1D oscillators with thermal noise fluctuation

We use the proposed clustering method to validate the decomposition of the synthetic signal into its original modes. We demonstrate the effectiveness of our method in dealing with continuous spectra by comparing it with the results of clustering eigenfunctions by DMD, which incorporate time delays embedded in the dictionary. In addition to the DMD-based method, we also applied the Measure-Preserving Extended Dynamic Mode Decomposition (mpEDMD) algorithm^22^ to analyze the data in this experiment. The mpEDMD algorithm is a structure-preserving data-driven approximation of Koopman operators for measure-preserving dynamical systems, which is guaranteed to converge to the correct spectral information. We included mpEDMD in our analysis to compare its performance with the other methods. The one-dimensional synthetic signal shown in the left panel of Fig. 1 is an observed signal $x (t)$ composed of signal 1 (as $x_1 (t)$) and signal 2 (as $x_2 (t)$), each with harmonic oscillations of different frequencies and thermal noise. This signal is governed by the following system of differential equations:

6 $$\begin{aligned} \begin{aligned}{}&\ddot{x}_1 (t) = - \omega _1 x_1(t) + \sigma _1 \eta _1 (t), \; \ddot{x}_2 (t) = - \omega _2 x_2(t) + \sigma _2 \eta _2 (t), \\&x (t) = x_1 (t) + x_2 (t), \end{aligned} \end{aligned}$$

where the frequencies $\omega _1, \omega _2$ are set to $0.06 \pi$ rad/s and $0.24 \pi$ rad/s, respectively, and the magnitude of the thermal noise $\sigma$ is set to 1.0. The qualitative behavior of signal 1 and signal 2 is different, as shown in the figure. We address the challenge of separating the original signals 1 and 2 from the synthetic signal. The signals are sampled at intervals of $\text {dt} = 0.1$ in the range $t = [0, 190]$. As a dictionary, we use the union of the Krylov subspace by time-delay embedding, a common method in DMD-type algorithms^23,24^. We construct the $M \times N$ matrices $\Psi _X$ and $\Psi _Y$ from the time series $(x_0, \ldots , x_n)$, using a single trajectory of length $M = 1900$ for the observation $g$ and a delayed embedding of $N = 900$,

7 $$\begin{aligned} g(x_t)&= (x_t, x_{t+1}, ...., x_{t+M-1})^T \in \mathbb {R}^M, \end{aligned}$$

8 $$\begin{aligned} \Psi _X&= \{ g(x_0), \mathcal {K} g(x_0), ...., \mathcal {K}^{N - 1} g(x_0) \} = \{ g(x_0), g(x_1), ..., g(x_{N-1}) \} \in \mathbb {R}^{M \times N}, \end{aligned}$$

9 $$\begin{aligned} \Psi _Y&= \{\mathcal {K} g(x_0), \mathcal {K}^2 g(x_0), ...., \mathcal {K}^{N} g(x_0) \} = \{ g(x_1), g(x_2), ..., g(x_{N}) \} \in \mathbb {R}^{M \times N}. \end{aligned}$$

The center of Fig. 1 shows the output of Algorithm 1. The pseudospectra (contours) and DMD eigenvalues (red dots) computed from the data are shown in the figure. The rank of $\Psi _X$ gives us 900 eigenvalues. The intensity of the color indicates the common logarithm of the residuals computed by ResDMD, with darker colors indicating smaller residuals. Some DMD eigenvalues have been identified as spurious modes due to their large residuals. These modes are unreliable eigenvalues that tend to change with small perturbations of the data matrix, indicating the presence of spectral pollution due to noise. The small residuals are found in the unit disk region. The results of the pseudospectra suggest that the system can be considered as a dynamical system with conservation of measure.

The right panel of Fig. 1 shows the DMD spectrum, where the horizontal axis represents the argument of the complex eigenvalues ($\lambda = e^{i \theta }$) and the vertical axis shows the absolute values of the DMD coefficients. The DMD spectrum is expected to show two broad peaks at $0.6 \pi \approx . 0.188$ rad/s and $0.24 \pi \approx . 0.754$ rad/s. However, the DMD spectrum showed scattered eigenvalues with significant coefficients and did not delineate the expected peaks. The frequencies of the two large-amplitude modes are $\theta \approx . 0.30, 1.20$ rad/s, indicating a bias toward the high-frequency side. Although there are large-amplitude modes in the high-frequency component of the DMD spectrum, they are thought to be influenced by spectral pollution due to the continuous spectrum of thermal noise^12^. The difficulty in identifying dominant modes is attributed to the spectral contamination observed in the center of Fig. 1, which poses a significant challenge to the analysis of real data in DMD^25^. In scenarios where spectral analysis via DMD proves challenging, we advocate the use of ResDMD for clustering analysis of continuous spectra.

**Figure 3:**
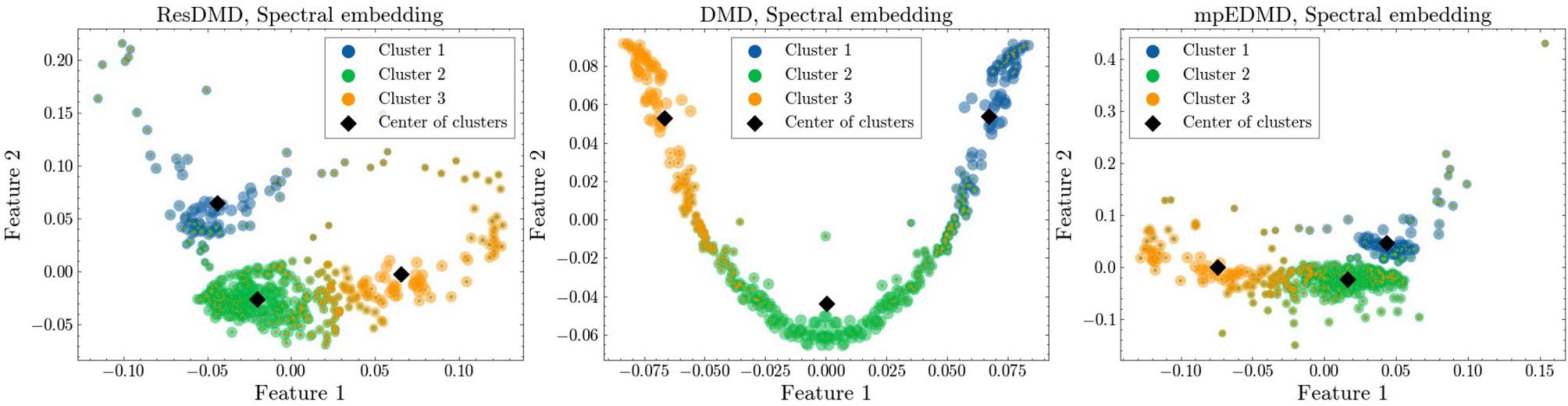
Spectral embedding of ResDMD’s pseudoeigenfunctions of principal angle (Left), DMD’s eigenfunctions of principal angle (Middle), mpEDMD’s eigenfunctions of principal angle (Right). We use fuzzy C-means to perform soft clustering, with the three clusters color-coded according to their membership degree.

**Figure 4:**
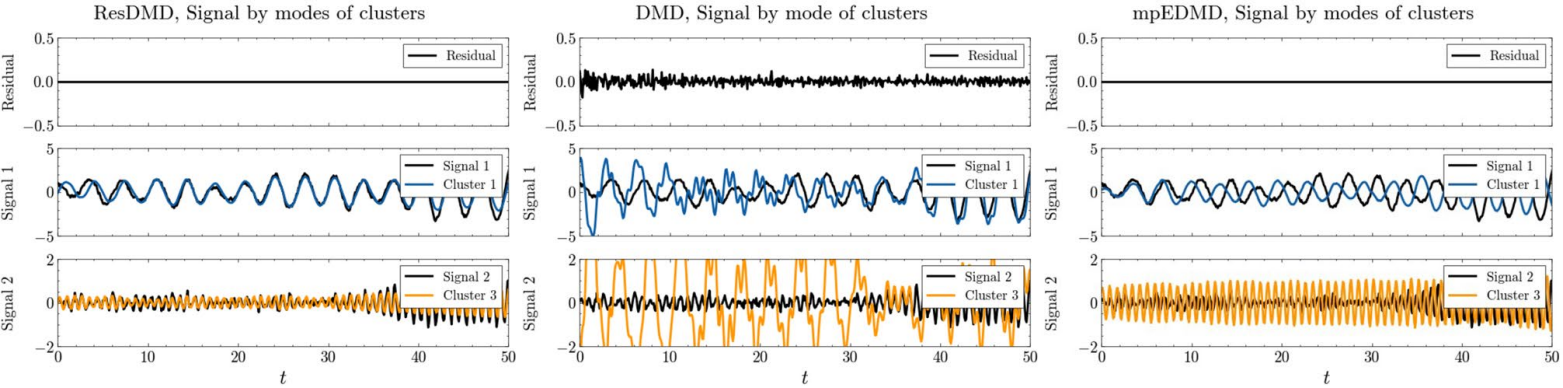
Results of the signal decomposition by clustering. Left: Reconstruction of the mode set by clustering the generalized eigenfunctions of ResDMD. The residual shows the error between the observed signal data and all modes before clustering in ResDMD. Signal 1 and Signal 2 correspond to those shown in the left panel of Fig. 1. The blue, and orange lines show the mode set reconstructions by clustering, respectively. Middle: Mode set reconstruction by clustering the eigenfunctions of DMD, plotted in the same way as on the left. Right: Mode set reconstruction by clustering the eigenfunctions of mpEDMD.

Figure 2 shows the spectral measures computed by ResDMD for the continuous spectra (left), the DMD eigenvalue spectra (middle), and the mpEDMD spectral measure (right). We set the smoothing parameter ($\varepsilon = 0.01$) for the approximation of the spectra by ResDMD in the left figure. The DMD spectra in the middle figure illustrate the frequency dependence of the absolute value of the DMD amplitudes. ResDMD’s smoothed spectral measure clearly shows two expected peaks, effectively separated by clustering. Each peak has a broad width and is resolved as a set of points. Clustering categorized the spectrum into broad spectra with peaks at ($\theta \approx 0.192$) rad/s and ($\theta \approx 0.761$) rad/s, as well as high-frequency components. Points in cluster 1 represent low-frequency spectra, while points in cluster 3 capture high-frequency spectra. Overlapping spectra indicate classification ambiguity.

The mpEDMD method, shown in the right figure of Fig. 2, approximates the spectral measure by assuming the Koopman operator is unitary. The mpEDMD spectral measure also exhibits two expected peaks, which clustering effectively separates. This demonstrates that mpEDMD can capture the essential features of the continuous spectrum by computing eigenfunctions under the assumption of a unitary Koopman operator^22^. The DMD spectrum in the middle figure shows a different clustering pattern compared to ResDMD and mpEDMD. Points in cluster 2 are at high frequencies with no identifiable peaks, corresponding to a long-tail distribution due to thermal noise. Interestingly, clustering of DMD eigenfunctions separates the high-frequency tail (cluster 2) from the other clusters. This suggests that these eigenfunctions may share properties that distinguish them from others. However, the DMD approach does not effectively classify the peaks, as clusters 1 and 3 are mixed. This highlights the significant impact of spectral contamination when using DMD eigenfunctions. The results suggest that pseudoeigenfunctions computed by ResDMD and eigenfunctions computed by mpEDMD (under the assumption of a unitary Koopman operator) are more suitable for dynamic feature extraction than those in DMD, especially when the analysis cannot be adequately described by a point spectrum containing noise. The separation of the high-frequency tail in the DMD results warrants further investigation into the properties of the associated eigenfunctions and their role in the system’s dynamics.

Figure 3 shows the embedding of the kernel principal angle using spectral clustering by fuzzy C-means for ResDMD’s pseudoeigenfunctions (left), DMD’s eigenfunctions (middle), and mpEDMD’s eigenfunctions (right). The feature space represents a low-dimensional embedding of the similarity matrix computed using the kernel principal angles between the eigenfunctions or pseudoeigenfunctions. For a detailed explanation of the clustering method, please refer to “Methods” section.

In the case of ResDMD, the clustering results in the feature space show a clear separation between the three clusters, corresponding to the low-frequency peak (Cluster 1), the high-frequency peak (Cluster 3), and the high-frequency tail (Cluster 2). Comparing the results of ResDMD and mpEDMD, we observe that in mpEDMD, Cluster 2 (green), which likely represents noise components at high frequencies, appears to be continuously connected with Cluster 3, which corresponds to the components of signal 2. In contrast, ResDMD shows a clear gap between these clusters, suggesting better differentiation of the dynamic structures. Additionally, in ResDMD, we observe data points that seem to be misclassified based on the spectral measure results in Fig. 1. When examining the feature space via spectral embedding, we find that data points are distributed in regions where the cluster membership is ambiguous, particularly around the [0.05, 0.10] area. This indicates the presence of pseudoeigenfunctions that do not belong to a single cluster, which likely contributes to misclassification. The fact that the dynamics possess continuous spectra suggests that there are pseudoeigenfunctions that cannot be distinctly classified into clusters. We use fuzzy C-means for soft clustering, where each data point is assigned to the cluster with the highest membership degree. However, ambiguous data points may be misclassified due to this method.

The DMD results (middle panel) show a distinct clustering pattern for the eigenfunctions. The high-frequency components corresponding to noise (Cluster 2) are continuously distributed and separated from Clusters 1 and 3. Interestingly, Cluster 2 is located in the region with smaller values of Feature 2, suggesting that Feature 2 represents a frequency-related characteristic. By characterizing noise as a high-frequency component, the clustering method for DMD eigenfunctions effectively separates the high-frequency tail from the other clusters. The mpEDMD results (right panel) exhibit a clustering pattern similar to that of ResDMD, with a clear separation between the three clusters in the feature space.

The spectral embedding and clustering results provide insights into the properties shared within each cluster and highlight the differences between the methods. ResDMD and mpEDMD demonstrate the ability to capture and separate the relevant dynamic features, while DMD eigenfunctions effectively isolate the high-frequency noise components.

Figure 4 shows the results of the signal decomposition by clustering. The left panel shows the reconstruction of the mode set by clustering the pseudoeigenfunctions of ResDMD. Signal 1 and signal 2 correspond to those shown in the left panel of Fig. 1. The blue and orange lines show the mode set reconstructions by clustering. The middle panel shows the mode set reconstruction by clustering the eigenfunctions of DMD, similar to the left panel. The right panel shows the mode set reconstruction by clustering the eigenfunctions of mpEDMD.

ResDMD demonstrates superior performance in clustering components that closely match the original modes compared to DMD and mpEDMD. The reconstructed signals from clusters 1 and 3 of ResDMD accurately capture the characteristics of signal 1 and signal 2, respectively, in terms of frequency and amplitude fluctuations. This highlights ResDMD’s ability to identify and separate the underlying dynamic features effectively. In contrast, DMD fails to reconstruct both signal 1 and signal 2 accurately. The reconstruction from cluster 1 of the DMD eigenfunctions contains high-frequency components that are not present in signal 1, and there are discrepancies in the amplitude. Similarly, the reconstruction from cluster 3 contains low-frequency oscillations similar to those in cluster 1, which are not present in signal 2. mpEDMD, on the other hand, manages to reconstruct signals close to signal 1 and signal 2, but with notable differences in phase and amplitude. This can be attributed to the assumption made by mpEDMD that the eigenvalues lie on the unit circle. In the presence of noise, the spectral characteristics might slightly deviate from the circle, leading to biases in the phase and amplitude when reconstructing signal data from the clustering results.

Comparing the residuals between the original synthetic signal and the reconstructed signal from all modes used to compute the spectral measures at the top of Fig. 4, it is evident that ResDMD achieves significantly lower residuals than DMD and mpEDMD. This demonstrates ResDMD’s ability to minimize spectral pollution and accurately reconstruct the original modes. These findings highlight the superiority of ResDMD in capturing and separating the relevant dynamic features, enabling accurate reconstruction of the original modes. DMD’s inability to reconstruct the signals and mpEDMD’s phase and amplitude biases underscore the importance of considering the effects of noise and the assumptions made by different methods when analyzing complex dynamical systems.

### Coupled Hénon maps

We analyze the phenomenon of coupled chaos synchronization as an example of complex dynamics. Synchronization in networks of coupled oscillators is ubiquitous in various scientific disciplines, including biology, physics, chemistry, and social networks^26^. The synchronization of chaotic systems is a rich phenomenon and a multidisciplinary topic with a wide range of applications^27–29^. The form of synchronization in chaotic systems varies depending on the nature of the interacting systems, the type of coupling, and the proximity between the systems. Full synchronization between identical systems, or phase synchronization, is a well-known example^30–32^. A significant advance in these synchronizations is generalized synchronization (GS), which is characterized by a time-independent nonlinear functional relationship between the states of two systems^33–36^. Although several data-driven methods have been proposed^36–38^, the experimental detection and characterization of GS from observational data remains challenging. We emphasize the utility and feasibility of Koopman eigenfunctions in both analytical and data-driven approaches to oscillator synchronization^39–41^.

**Figure 5:**
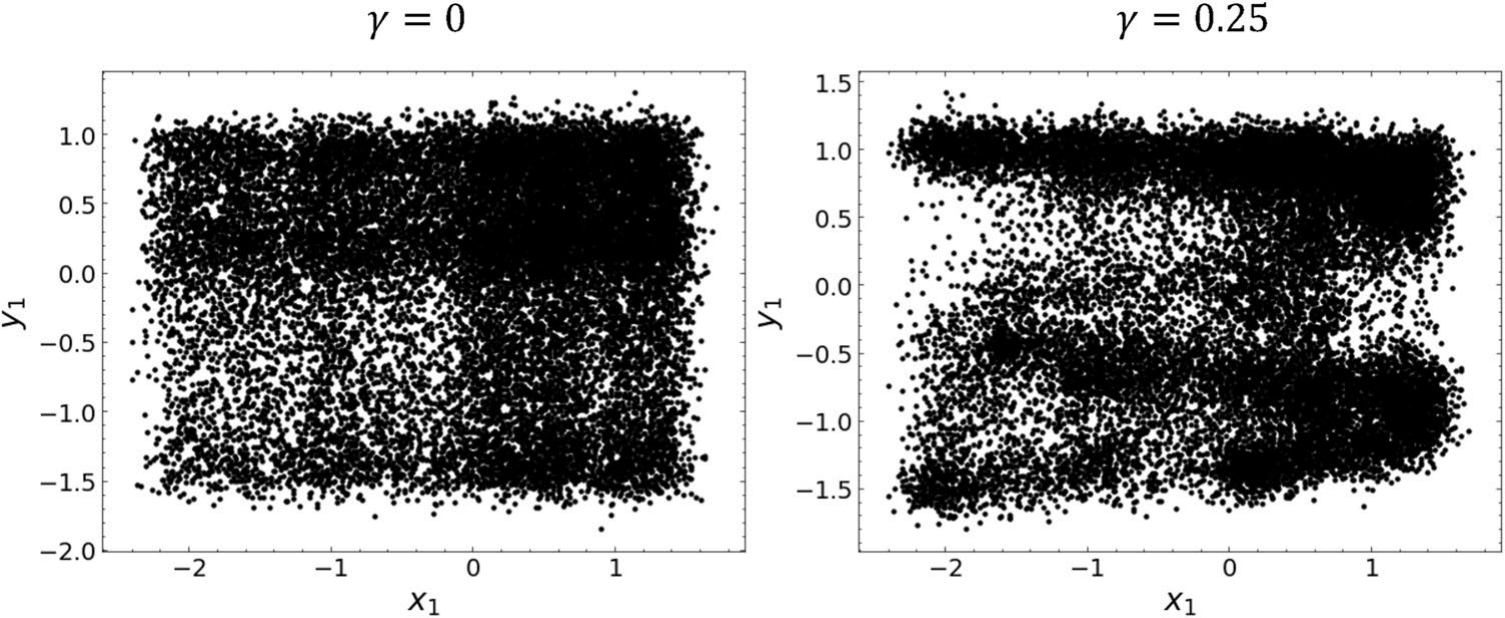
Projections of strange attractors of the coupled Hénon map onto the $(x_1, y_1)$ plane. Left: $\gamma = 0.0$. Right: $\gamma = 0.25$.

**Figure 6:**
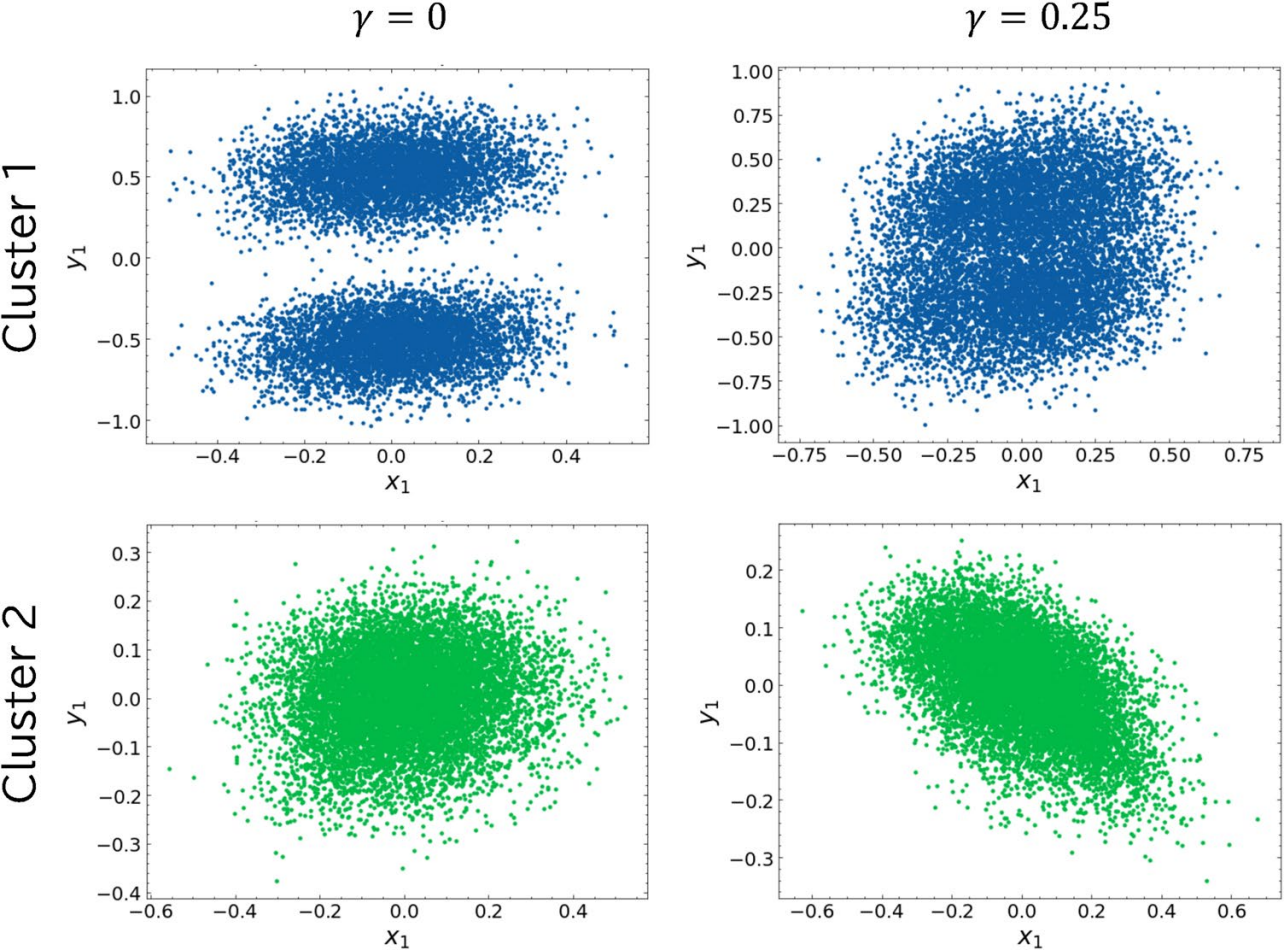
Results of the coupled Hénon map projections onto $(x_1, y_1)$ by our clustering method. The number of clusters is 2, indicating a low-dimensional representation of chaos. Left and right correspond to $\gamma = 0$ and $\gamma = 0.25$, respectively; top and bottom refer to the number of clusters.

As the fundamental chaos map inducing GS, we study the two unidirectionally coupled Hénon maps governed by the following difference equations^42^:

10 $$\begin{aligned} \begin{aligned}{}&X:\left\{ \begin{array}{l} x_1(t+1)=1.4-x_1(t)^2+0.3x_2(t) \\ x_2(t+1)=x_1(t) \end{array}\right. , \\&Y:\left\{ \begin{array}{l} y_1(t+1)=1.4-\left\{ \gamma x_1(t)y_1(t)+(1-\gamma )y_1(t)^2\right\} +0.1y_2(t) \\ y_2(t+1)=y_1(t) \end{array}\right. , \end{aligned} \end{aligned}$$

where $\gamma$ is the coupling strength between two systems $X$ and $Y$. Figure 5 shows the projections of $x_1$ and $y_1$ of the coupled Hénon maps onto the $(x_1, y_1)$ plane (left: $\gamma =0$, right: $\gamma =0.25$). We generated this time series of 30,000 points with $\text {dt} = 1$. It is clear that when the coupling strength $\gamma$ is large, a highly nonlinear and complex response relationship forms between two different chaotic dynamics. Previous studies have detected GS in coupled Hénon maps by examining the correlation between the dynamics of the two systems through various nonlinear transformations^34,36^. Given the apparent differences in the attractors, we aim to derive a representation that characterizes GS from the attractors. Periodic chaos, such as Hénon maps, can have continuous spectra and their pseudoeigenfunctions as dynamical features^43^.

The discrete components identified by the clustering methodology correspond to different states of the chaotic system. In this example, we considered a scenario with two different chaotic maps being observed simultaneously, each with its continuous spectrum. These chaotic maps are coupled with a coupling constant $\gamma$. When $\gamma = 0$, the two dynamics are completely independent, while for $\gamma = 0.25$, the chaotic maps exhibit generalized synchronization (GS) as described in Ref.^36^. By clustering the pseudoeigenfunctions reflecting the spectral properties of the dynamics, we can identify discrete states in terms of their spectral characteristics. This allows us to distinguish between different dynamical states even in a spectrally continuous system. Here, assuming that the two chaotic systems are coupled, we selected two clusters from the two-dimensional observations $x_1$ and $y_1$ to match the observation dimensions. The clusters identified by our proposed method are decompositions of the continuous spectrum into sets of modes whose pseudoeigenfunctions are closely related. Since this can be seen as a reduction of dynamics with chaotic features, we investigate whether GS can be detected and characterized by analyzing these clusters. As in “1D oscillators with thermal noise fluctuation” section, we construct the $M \times 2N$ matrices $\Psi _X$ and $\Psi _Y$ from the time series $(x_1(0), \ldots , x_1(n))$ and $(y_1(0), \ldots , y_1(n))$, using trajectories of length $M = 10,000$ as observations $g$ and a delayed embedding of $N = 500$,

11 $$\begin{aligned} g_1&= g(x_1(t)) = \left( x_1(t), x_1(t+1), \ldots , x_1(t+M-1)\right) ^T \in \mathbb {R}^M, \end{aligned}$$

12 $$\begin{aligned} g_2&= g(y_1(t)) = \left( y_1(t), y_1(t+1), \ldots , y_1(t+M-1)\right) ^T \in \mathbb {R}^M, \end{aligned}$$

13 $$\begin{aligned} \Psi _X&= \{ g_1, \mathcal {K} g_1, \ldots , \mathcal {K}^{N-1} g_1, g_2, \mathcal {K} g_2, \ldots , \mathcal {K}^{N-1} g_2 \} \in \mathbb {R}^{M \times 2N}, \end{aligned}$$

14 $$\begin{aligned} \Psi _Y&= \{ \mathcal {K} g_1, \mathcal {K}^2 g_1, \ldots , \mathcal {K}^{N} g_1, \mathcal {K} g_2, \mathcal {K}^2 g_2, \ldots , \mathcal {K}^{N} g_2 \} \in \mathbb {R}^{M \times 2N}. \end{aligned}$$

We computed and analyzed the ResDMD matrices $\tilde{G}$, $\tilde{A}$, $\tilde{L}$ from the $10,000 \times 1000$ matrix generated by the simulation.

Figure 6 shows the results of the decomposition into 2 clusters and their reconstruction into the original coordinate system for each cluster. The clustering results for $\gamma =0$ and $\gamma =0.25$ are shown in the left and right panels, respectively. For each coupling parameter, the top and bottom panels show the clusters; cluster 1 (blue) is shown at the top, and cluster 2 (green) is shown at the bottom. For $\gamma =0$, two distinct clusters were observed within cluster 1, while for $\gamma =0.25$ the clusters appeared to converge. This result illustrates that $\gamma = 0$ (without coupling) and $\gamma = 0.25$ (with coupling) have distinctly different dynamics. The results further indicate that coupling causes two independent attractors to converge and behave as a single attractor. In cluster 2, no correlation between $x_1$ and $y_1$ was observed for $\gamma =0$, while a clear correlation was observed for $\gamma =0.25$. Generalized Synchronization (GS) implies that $x_1$ and $y_1$ are correlated by nonlinear transformations. The Cluster 2 result at $\gamma = 0.25$, where this correlation is evident, serves as evidence for GS.

## Discussion

To advance data-driven analysis using Koopman operators, we have developed a method to analyze dynamics with an aperiodic structure. Using the analogy between subspaces for pseudoeigenfunctions, we have implemented clustering in spaces capable of representing continuous spectra. Our algorithm incorporates the principles of spectral clustering and fuzzy C-means. Spectral clustering is a well-established and widely applied technique, while fuzzy C-means provides a soft clustering approach that allows the representation of the degree of overlap between clusters. The strengths of our method lie in its flexibility, versatility, and ease of implementation. It reveals the intrinsic dynamical structure within the data by decomposing complex dynamics into a collection of modes characterized by a continuous spectrum.

In the analysis of one-dimensional (1D) oscillators with thermal noise fluctuations, our method was successful in delineating the structure of the dynamics, whereas DMD and mpEDMD were not. By examining a simple one-dimensional synthetic signal, we demonstrate that spectral analysis goes beyond the conventional eigenvalue decomposition typically associated with DMD. Thermal noise leads to spectral broadening, which confounds the accurate estimation of the spectrum by DMD eigenvalues. From the pseudospectra computed by ResDMD, we identified several spurious eigenvalues with significant residuals, which we believe negatively affect the estimation^18^. In contrast, the continuous spectrum derived from the ResDMD matrices showed the expected double peak structures. We were able to isolate the original two signals by clustering the pseudoeigenfunctions, which was not possible by clustering the DMD eigenfunctions.

It should be noted that mpEDMD performs better than DMD for the proposed clustering. While mpEDMD was successful in capturing the continuous spectrum and enabling clustering, it struggled to accurately reconstruct the original modes due to its assumption of a unitary Koopman operator. The presence of noise caused deviations from the unit circle in the spectral characteristics, leading to biases in the phase and amplitude of the reconstructed signals. This highlights the importance of considering the effects of noise on the assumptions made by different methods.

In addition to the clustering results of the spectral measures, we verified that our proposed method can segment the synthetic signal into two components with distinct peaks and a high-frequency noise component. We conducted spectral analysis to characterize this noise component and support the assumption that it is Brownian noise. In the case of Brownian noise, the spectrum is continuous and exhibits an inverse square relationship with frequency. Specifically, as the frequency increases, the characteristics of the harmonic oscillation peaks diminish and the long-tail component of Brownian noise becomes dominant. The spectra identified in the clusters exhibit a long-tailed distribution and show behavior consistent with an inverse square relationship with frequency. These findings align with the characteristics of Brownian noise. Therefore, based on the results of our spectral analysis and the fact that the data was synthesized using thermal noise, we concluded that this noise component is Brownian noise^44^. These results show that continuous spectra can represent signals affected by thermal noise and that the corresponding pseudoeigenfunctions have dynamic properties. The dynamic properties mean oscillatory and phase behaviors that appear structured, yet irregular, reflecting the inherent dynamics of the system. Clustering these pseudoeigenfunctions facilitates the decomposition of the synthetic signal into its constituent components, including the noise component.

The clustering results of the spectral measures and the reconstruction of the original modes demonstrate the superiority of ResDMD in capturing and separating the relevant dynamic features. ResDMD’s ability to minimize spectral pollution enables more accurate reconstruction of the original modes compared to DMD and mpEDMD, as evidenced by the significantly lower residuals. These findings underscore the importance of considering the effects of noise and the assumptions made by different methods when analyzing complex dynamical systems.

In the analysis of coupled Hénon maps, our clustering method successfully identifies qualitative changes in complex dynamics. Coupled Hénon maps, among the most fundamental systems that exhibit coupled chaos, lead to generalized synchronization (GS) when there is a significant coupling strength^36,42^. Using our method to classify the coupled Hénon maps into two clusters, we reconstruct the original two-dimensional dynamics and observe marked differences between states with and without GS. A qualitative assessment of the reconstructed dynamics for each cluster reveals that GS causes originally distinct attractors to converge, along with an increase in the correlation between variables indicative of GS. These results suggest that even in complex dynamics, such as chaos, the simplified representation achieved by clustering pseudoeigenfunctions provides a solid basis for characterizing the state of the system.

In this study, we introduced a clustering method for pseudoeigenfunctions and, through computational experiments, proposed a new analytical direction: the decomposition into mode sets, unattainable with traditional DMD, and its application to the extraction of bases describing chaos. The data-driven algorithm we developed facilitates the comparison of eigenfunctions using subspaces. However, our method is not without limitations. First, it requires that the dynamical system be measure-preserving when computing smoothed approximate spectral measures with the ResDMD matrices. Depending on the dynamics, the analysis may require a sufficiently long time series to ensure that it is measure-preserving. Second, determining the optimal number of clusters is a challenge. In this research, we approximated the number of clusters by observing the variation in the eigenvalues of the Laplacian from spectral clustering, a heuristic approach with minimal theoretical support (we fixed the number of clusters at 2 for chaos analysis). We used a toy model to demonstrate the effectiveness of our proposed method. Extending this method to real data, investigating conditions under which clustering may fail, and estimating the parameters of continuous spectra are areas for future research. Another promising direction for future work is to extend our method to larger state-space dimensions. As discussed in Refs.^12,45^, combining ResDMD with kernel methods has the potential to enable the clustering of pseudoeigenfunctions in high-dimensional systems. Kernel methods allow for implicit computation in high-dimensional feature spaces by using kernel functions to calculate similarities between data points, reducing the computational burden when dealing with high-dimensional data. Exploring the relationship between this kernel-based approach and functional space theory could provide valuable insights into the behavior of complex dynamical systems.

In conclusion, the clustering of pseudoeigenfunctions computed by ResDMD offers a powerful tool for analyzing dynamical systems with continuous spectra. ResDMD does not require the assumption of a unitary Koopman operator, making it applicable to a wider range of dynamical systems.

Recent advancements in the field, such as the Rigged Dynamic Mode Decomposition (Rigged DMD) introduced by Colbrook et al.^46^, offer potential improvements for measure-preserving systems. Rigged DMD extends the capabilities of spectral analysis by providing a framework for computing generalized eigenfunction decompositions of Koopman operators, even in the presence of continuous spectra. It is based on mpEDMD, which assumes a unitary Koopman operator. While this assumption limits its applicability to measure-preserving systems, Rigged DMD provides smoothed approximations of generalized eigenfunctions that more accurately reflect spectral characteristics compared to the pseudoeigenfunctions computed by ResDMD.

It is important to note the complementary nature of ResDMD and Rigged DMD. For measure-preserving systems, Rigged DMD can improve the handling of continuous spectra and lead to more accurate decompositions of complex dynamical systems through its generalized eigenfunction approach. However, for systems where the measure-preserving property cannot be assumed, our ResDMD-based method remains a valuable and effective approach.

For future research, we envision applying our clustering methodology to the generalized eigenfunctions computed by Rigged DMD for measure-preserving systems. This approach is expected to enable for more robust and accurate clustering based on spectral characteristics. By leveraging the strengths of Rigged DMD in handling continuous spectra and providing more accurate generalized eigenfunction decompositions, we anticipate achieving a more precise analysis of complex dynamical systems, especially those with intricate spectral properties.

Additionally, extending our method to larger state-space dimensions remains an important goal. Combining spectral decomposition techniques with kernel methods has the potential to enable the clustering of generalized eigenfunctions in high-dimensional systems. Investigating the relationship between these spectral decomposition techniques, kernel-based approaches, and functional space theory could provide valuable insights into the behavior of complex dynamical systems and further expand the applicability of our method.

By following these research directions, we can develop a more comprehensive toolkit to analyze a wide range of dynamical systems. This approach will allow for more robust and widely applicable methods in the study of dynamical systems with continuous spectra, ultimately bridging the gap between theoretical advancements and practical applications in the field of dynamical systems analysis.

## Methods

In this study, we develop a data-driven methodology for analyzing aperiodic dynamics caused by noise or chaos. We present a spectral analysis method that captures data aperiodicity by clustering pseudo-eigenfunctions associated with continuous spectra, derived using ResDMD^12,13^.

In this section, we outline ResDMD algorithm for computing pseudo-eigenfunctions and continuous spectra, followed by a discussion of clustering these functions. To lay the groundwork for ResDMD, we first describe the Extended Dynamic Mode Decomposition (EDMD) framework for constructing matrices from data, which approximates the Koopman operator. We then discuss ResDMD, an adaptation of EDMD that facilitates the construction of spectral properties by computing pseudospectra and pseudoeigenfunctions from the data. Finally, we discuss spectral clustering, which incorporates fuzzy C-means to identify data elements that represent the characteristics of the dynamics.

### Extended dynamic mode decomposition (EDMD)

EDMD is a well-established technique for approximating Koopman operators $\mathcal {K}$ by a finite matrix $K \in \mathbb {C}^{N \times N}$, which is crucial for elucidating complex dynamical systems. It analyzes data to reveal the underlying patterns and forms the basis of ResDMD method.

Our data-driven approach uses a dataset of snapshots $\{ \varvec{x}^{(m)}, \varvec{y}^{(m)} = F(\varvec{x}^{(m)})\}_{m=1}^M$ and a dictionary of observables $\{\psi _1, \ldots , \psi _N \}$ in space $L^2 (\Omega , \omega )$. The choice of dictionary varies, and for DMD-type algorithms we opt for delay-embedding of observables, a widely used technique^3^. EDMD chooses a matrix $K$ that best approximates $\mathcal {K}$ within a finite-dimensional subspace $V_N = \textrm{span} \{\psi _1, \ldots , \psi _N\}$. Let $\mathcal {P}_{V_N}$ be the orthogonal projection onto $V_N$, where $K$ is intended to approximate a matrix representation of $\mathcal {P}_{V_N} \mathcal {K} \mathcal {P}_{V_N}^*$ if enough data is available. Within a Galerkin framework, we have

15 $$\begin{aligned} \langle \mathcal {K} \psi _k, \psi _j \rangle = \sum _{i=1}^N K_{i,j} \langle \psi _i, \psi _j \rangle , \quad 1 \le j, k \le N, \end{aligned}$$

where $K_{i, j}$ is the $(i, j)$ component of the matrix $K$. An explicit solution of this equation is given by

16 $$\begin{aligned} K = G^{\dagger } A \approx \tilde{G}^{\dagger } \tilde{A} = (\Psi _X^* W \Psi _X)^{\dagger } (\Psi _X^* W \Psi _Y), \quad G_{i,j} = \langle \psi _i, \psi _j \rangle , \; A_{i,j} = \langle \mathcal {K} \psi _i, \psi _j \rangle , \end{aligned}$$

where $\dagger$ denotes the pseudoinverse and $W$ is the diagonal matrix containing the quadrature weights ${w_m}$. The elements $G_{i,j}$ and $A_{i,j}$ correspond to the ${i,j}$ entries of the matrices $G$ and $A$, respectively. The weights ${w_m}$ indicate the importance given to each snapshot. We assume $w_m = 1/M$ for the random sampling^9,47^. The feature map $\Psi$ is defined by aligning $\psi$ in the row direction:

17 $$\begin{aligned} \Psi (\varvec{x}) = \left[ \psi _1(\varvec{x}), \psi _2(\varvec{x}), \ldots , \psi _N(\varvec{x})\right] \in \mathbb {C}^{1 \times N}. \end{aligned}$$

Then any function $g \in V_N$ can be written as $g (\varvec{x}) = \sum _{j=1}^{N} \psi _j (\varvec{x}) g_j = \Psi (\varvec{x})\varvec{g}$ for a given vector $\varvec{g} \in \mathbb {C}^N$. Using the matrix $K$ it follows that

18 $$\begin{aligned}{}[\mathcal {K} g] (\varvec{x}) \approx \sum _{i=1}^N \left( \sum _{j=1}^N K_{i,j} g_j\right) \psi _i (\varvec{x})= \Psi (\varvec{x}) (K \varvec{g}). \end{aligned}$$

The matrices $\Psi _X$ and $\Psi _Y$, each of size $M \times N$, are defined as

19 $$\begin{aligned} \Psi _X&=\begin{pmatrix} \Psi \left( \varvec{x}^{(1)}\right) \\ \vdots \\ \Psi \left( \varvec{x}^{(M)}\right) \end{pmatrix} \in \mathbb {C}^{M \times N}, \quad \Psi _Y=\begin{pmatrix} \Psi \left( \varvec{y}^{(1)}\right) \\ \vdots \\ \Psi \left( \varvec{y}^{(M)}\right) \end{pmatrix} \in \mathbb {C}^{M \times N}. \end{aligned}$$

The elements of the matrices $G$ and $A$, which represent inner products, must be approximated using snapshot data as described. Let $\tilde{G}$ and $\tilde{A}$ denote the numerical approximations:

20 $$\begin{aligned} \tilde{G}_{jk} = \sum _{m=1}^M w_m \psi _k\left( \varvec{x}^{(m)}\right) \overline{\psi _j\left( \varvec{x}^{(m)}\right) }&= \Psi _X^* W \Psi _X \approx \langle \psi _k, \psi _j\rangle = G_{jk}, \end{aligned}$$

21 $$\begin{aligned} \tilde{A}_{jk} = \sum _{m=1}^M w_m \psi _k\left( \varvec{y}^{(m)}\right) \overline{\psi _j\left( \varvec{x}^{(m)}\right) }&= \Psi _X^* W \Psi _Y \approx \langle \mathcal {K} \psi _k, \psi _j\rangle = A_{jk}. \end{aligned}$$

In the limit of large data sets, such as $M \rightarrow \infty$, the eigenvalues derived by EDMD converge to the spectrum of $\mathcal {P}_{V_N} \mathcal {K} \mathcal {P}_{V_N}^*$. Consequently, the eigenvalue approximation of the spectrum of $\mathcal {K}$, $\sigma _p (\mathcal {K})$, is closely related to the so-called finite section method^48^. Since the finite section method is prone to spectral pollution, the establishment of an independent mechanism for verifying the accuracy of the proposed eigenvalue-eigenvector pairs becomes crucial. ResDMD emerges as a strategy to circumvent spectral pollution and compute pseudospectra.

The pseudospectra of an operator are the set of values that closely approximate the spectrum of the operator^18,49^. ResDMD facilitates the computation of pseudospectra using EDMD matrices, providing a data-driven perspective on the spectrum of Koopman operators that includes continuous spectra.

### Residual dynamic mode decomposition (ResDMD)

**Algorithm 1:**
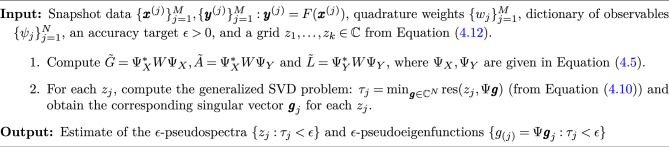
ResDMD for pseudospectra^12^

ResDMD includes modifications to facilitate a more detailed analysis of continuous spectra in dynamical systems. It is uniquely suited to address the challenges posed by infinite-dimensional operators, which are often encountered in data characterized by chaos and noise. The core of ResDMD is its ability to handle residuals associated with these infinite-dimensional operators. To compute residuals, ResDMD uses an additional matrix $\mathcal {K}^* \mathcal {K}$, which is different from traditional DMD approaches, and establishes an error limit. For each potential eigenpair $(\lambda , g)$, where $\lambda \in \mathbb {C}$ and $g \in V_N$, the degree of reliability for that eigenpair can be assessed by calculating the following relative squared residual:

22 $$\begin{aligned} \begin{aligned} \frac{\int _{\Omega }|[\mathcal {K} g](\varvec{x})-\lambda g(\varvec{x})|^2 d \omega (\varvec{x})}{\int _{\Omega }|g(\varvec{x})|^2 d \omega (\varvec{x})} = \frac{\langle (\mathcal {K}-\lambda ) g,(\mathcal {K}-\lambda ) g\rangle }{\langle g, g\rangle } = \frac{\langle \mathcal {K} g, \mathcal {K} g\rangle - \lambda \langle g, \mathcal {K} g\rangle - \bar{\lambda } \langle \mathcal {K} g, g\rangle + |\lambda |^2 \langle g, g\rangle }{\langle g, g\rangle }. \end{aligned} \end{aligned}$$

If the residual is sufficiently small, $\lambda$ can be considered as an approximation of the spectrum of a normal operator $\mathcal {K}$, where $g$ is the corresponding eigenfunction. For non-normal $\mathcal {K}$, which may have continuous spectra, the residual still provides a measure of accuracy. When $\lambda$ is part of a dense subspace where residuals in the complex plane are minimal, it means a continuous spectrum.

The concept of residuals is closely related to pseudospectra^49^. Pseudospectra, defined by the norm of the resolvent in spectral theory, is considered to be a generalization of eigenvalues^19,50,51^.

#### Definition 1

For any $\lambda \in \mathbb {C}$ and a relative tolerance $1> \epsilon > 0$, the approximate point $\epsilon$-pseudospectra is defined as:

23 $$\begin{aligned} \sigma _\epsilon (\mathcal {K})=\textrm{U}_{|E|<\epsilon } \sigma (\mathcal {K}+E)=\left\{ \lambda \in \mathbb {C}: \limsup _{M \rightarrow \infty }\Vert {(\mathcal {K} - \lambda I)^{-1}}\Vert ^{-1} < \epsilon , \;\; \Vert {g}\Vert = 1 \right\} . \end{aligned}$$

This formula denotes a set of $\lambda$ which is resistant to perturbations by all bounded operators $E$ with a norm strictly less than $\epsilon$. In this context, $g$ is called a pseudoeigenfunction if there exists $\lambda \in \mathbb {C}$ such that the residual in Eq. (22) is bounded by a small $\epsilon$.

Now, we present the numerical approximation:

24 $$\begin{aligned}{}[\textrm{res} (\lambda , g)]^2&= \frac{\sum _{j, k=1}^{N} \overline{g_j} g_k\left[ \left( \Psi _Y^* W \Psi _Y\right) _{j k}-\lambda \left( \Psi _Y^* W \Psi _X\right) _{j k}-\bar{\lambda }\left( \Psi _X^* W \Psi _Y\right) _{j k}+|\lambda |^2\left( \Psi _X^* W \Psi _X\right) _{j k}\right] }{\sum _{j, k=1}^{N} \overline{g_j} g_k\left( \Psi _X^* W \Psi _X\right) _{j k}} \nonumber \\&= \frac{\varvec{g}^*\left[ \tilde{L}-\lambda \tilde{A}^*-\bar{\lambda } \tilde{A}+\left| \lambda \right| ^2 \tilde{G}\right] \varvec{g}}{\varvec{g}^* \tilde{G} \varvec{g}}, \end{aligned}$$

where $\tilde{G}, \tilde{A}$ are the numerical approximations of EDMD detailed in Eqs. (20) and (21), and $\varvec{g} \in \mathbb {C}^N$ is a vector associated with pseudoeigenfunctions: $g = \Psi \varvec{g} \in V_N$. The matrix $\tilde{L}$ is defined as:

25 $$\begin{aligned} \tilde{L}_{jk} = \sum _{m=1}^M w_m \psi _k\left( \varvec{y}^{(m)}\right) \overline{\psi _j\left( \varvec{y}^{(m)}\right) } = \Psi _Y^* W \Psi _Y \approx \langle \mathcal {K} \psi _k, \mathcal {K}\psi _j\rangle = L_{jk}, \end{aligned}$$

which is actually an approximation of $\mathcal {K}^* \mathcal {K}$. Algorithm 1 provides practical approximations of the $\epsilon$ pseudospectra with strict convergence guarantees. We define the function $\tau _{M, N} (\lambda ) = \min _{\varvec{g}\in \mathbb {C}^N} \textrm{res} (\lambda , \Psi \varvec{g})$ within Algorithm 1. To analyze continuous spectra derived from pseudospectra, it is critical to estimate the narrowband portion from the approximate points. Since the pseudospectra $\sigma _\epsilon (\lambda )$ are continuous over $\lambda$, according to Dini’s theorem^52^, $\lim _{M \rightarrow \infty } \tau _{M, N}$ uniformly converges to $\sigma _\epsilon$ in compact subsets of $\mathbb {C}$. Suppose ${\text {Grid}}(N)$ is a sequence of finite grids which ensures that for any $\lambda \in \mathbb {C}$, $\lim _{N \rightarrow \infty } \textrm{dist} \left( \lambda , {\text {Grid}}(N)\right) = 0$. Based on previous research^13^, we assume that

26 $$\begin{aligned} {\text {Grid}}(N)=\frac{1}{N}[\mathbb {Z}+i \mathbb {Z}] \cap \{z \in \mathbb {C}:|z| \le N\}. \end{aligned}$$

For this investigation, the estimation used this grid, choosing $N$ large enough to ensure a general approximation of the continuous spectra. Since the pseudospectra asymptotically matches the continuous spectra, the associated pseudoeigenfunction provides an approximation of the spectral properties described by pseudoeigenfunctions.

The clustering is performed on the pseudoeigenfunctions derived from ResDMD, and we visualize the results on spectral measures. For visualization, it is more convenient to consider the corresponding probability measures $\nu _g$, which are defined on the periodic interval $[- \pi , \pi ]$ after transforming the variable $\lambda = e^{i \theta }$, thus $d \mu _g(\lambda ) = d \nu _g(\theta )$. We base our calculations on the assumption of a measure-preserving dynamical system for spectral measure analysis. It is recognized that the Fourier transform defines the integral transform in such a way that a finite interval is not required to compute a continuous spectrum from a Fourier series. In parallel, for the unit disk in the complex plane, the Cauchy transform is considered along with the Poisson kernel in the range $[- \pi , \pi ]$^53^. To compute the smoothed spectral measure $\nu _g^{\varepsilon }$, we approximate $\nu _g$ by convolution, defined as

27 $$\begin{aligned} \nu _g^{\varepsilon } (\theta _0)=\int \limits _{[-\pi , \pi ]_{\textrm{per}}} K_{\varepsilon } (\theta _0-\theta ) d \nu _g(\theta ), \;\; \text {where} \; K_{\varepsilon } (\theta )=\frac{1}{2 \pi } \frac{(1+\varepsilon )^2-1}{1+(1+\varepsilon )^2-2(1+\varepsilon ) \cos (\theta )}, \end{aligned}$$

with $\varepsilon$ as the smoothing parameter varying from 0 to 1. To improve the localization properties of the spectra, we used a high-order Poisson kernel. Colbrook et al. in a previous study^12^ provided a general definition for a $m$-th order kernel, giving a $m$-th order rate of convergence of $\nu _g^{\varepsilon }$ to $\nu _g$ both weakly and pointwise. Furthermore, their algorithm was used to compute smoothed approximations of spectral measures from the ResDMD matrices $\tilde{G}, \tilde{A}, \tilde{L}$ using the resolvent operator.

Analyzing the pseudospectra provides critical insight into the behavior of the system, in particular its resilience to perturbations and the stability of its dynamics. ResDMD is particularly useful for systems characterized by continuous spectra, where conventional methods fail to provide a clear understanding.

### Clustering pseudoeigenfunctions

**Algorithm 2:**
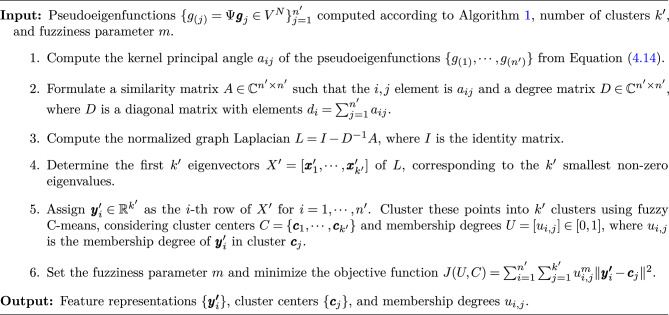
Classification of Eigenfunctions in subspaces

Our method incorporates graph clustering and constructs a similarity matrix from ResDMD’s pseudoeigenfunctions. This approach enables the identification and categorization of dynamic structures within the data, providing insights beyond those achievable with traditional DMD methods. To elucidate the structure of continuous spectra in the data, we introduce a clustering analysis aimed at classifying dynamics based on these spectra. Assuming that the dynamics consist of several distinct categories, we focus on pseudoeigenfunctions from ResDMD that reveal the characteristics of the dynamics associated with continuous spectra and propose clustering based on the similarity among these pseudoeigenfunctions.

To compute the pairwise similarity between pseudoeigenfunctions in the function space, we use the kernel principal angle^54^. This metric was chosen to quantify the similarity between the sets of pseudoeigenfunctions derived from the spectral properties computed by ResDMD. By applying the kernel principal angle to the subspaces spanned by the pseudoeigenfunctions, we can effectively identify subsets with similar spectral characteristics. This generalizes existing metrics used in time series models, such as the Martin distance for ARMA models and the distance based on subspace angles for linear state-space models^55–57^. This metric has also been used in previous studies to calculate the similarity of DMD modes^20^. By extending these metrics through the use of ResDMD and the kernel principal angle, we provide a unified framework for comparing a wide range of dynamical systems. Its ability to capture nonlinear relationships makes it particularly suitable for our analysis. To determine the inner product between the pseudoeigenfunctions $g_{(i)}$ and $g_{(j)}$, we address the generalized eigenvalue problem:

28 $$\begin{aligned} \left( \begin{array}{cc} 0 &{} \left( g_{(i)}^* g_{(j)}\right) ^* \\ g_{(i)}^* g_{(j)} &{} 0 \end{array}\right) \varvec{v} = a_{i j} \left( \begin{array}{cc} g_{(j)}^* g_{(j)} &{} 0 \\ 0 &{} g_{(i)}^* g_{(i)} \end{array}\right) \varvec{v}, \end{aligned}$$

where $a_{i j} \in \mathbb {C}$ are the eigenvalues corresponding to the principal angle between $g_{(i)}$ and $g_{(j)}$, and $\varvec{v}$ is an eigenvector associated with $a_{ij}$. The eigenvalues $a_{i j}$ quantify the similarity between the pseudoeigenfunctions represented by $g_{(i)}$ and $g_{(j)}$, where smaller angles indicate greater similarity.

The matrix formed by the kernel principal angles between each pair of components serves as the similarity matrix. To efficiently perform clustering with this matrix, spectral clustering is used, a method widely used in various disciplines for grouping data points that share similar characteristics. By using the eigenvectors of the graph Laplacian calculated from the similarity matrix provided by the kernel principal angle, we can map the data to a lower-dimensional space that reflects the similarity structure. Spectral clustering is robust to noise and perturbations and captures nonlinear manifold structures, making it suitable for our data^21^. Here, the similarity matrix acts as the weights of an undirected graph, with the pseudoeigenfunctions acting as vertices. To partition the graph while minimizing cuts and preserving similarity, a normalized graph Laplacian is used to solve the eigenvalue problem^58,59^. The Laplacian is computed as

29 $$\begin{aligned} L = I - D^{-1}A, \end{aligned}$$

where $I$ is the identity matrix, $A$ is the similarity matrix with elements $a_{ij}$, and $D$ is a degree matrix with elements $d_i = \sum ^{n'}_{j=1} a_{ij}$. Within this framework, the smallest eigenvalue is typically close to zero (except for numerical inaccuracies), indicating an intact graph. As detailed in Algorithm 2, selecting the eigenvectors associated with the smallest eigenvalues allows the similarity matrix to be embedded in a reduced-dimensional feature space. This strategy includes soft clustering in this feature space to deal with cases where continuous spectra overlap and cannot be separated. In particular, the fuzzy C-means algorithm is applied^60^. Given the continuous spectrum and the possibility that subsets with similar spectral properties may not have clear boundaries, we chose fuzzy C-means. It accommodates the possibility that the data points may belong to multiple clusters, reflecting the inherent ambiguity in the data^61^. This approach is beneficial for handling the overlapping nature of spectral features in the pseudoeigenfunctions. The whole procedure is described in Algorithm 2.

We also considered other clustering methods such as K-means and Multi-Dimensional Scaling (MDS). Although computationally efficient and simple, K-means clustering was less accurate for pseudoeigenfunctions with nonlinear characteristics. K-means assume that clusters are spherical and equally sized, which is not suitable for our data, which are characterized by nonlinear relationships. When applied to our pseudoeigenfunctions, K-means failed to accurately capture the complex, nonlinear structures, resulting in poor clustering performance. Multidimensional scaling (MDS) embeds the data in a lower dimensional space based on a distance matrix^62^. However, MDS relies on Euclidean distances, which do not effectively capture the nonlinear relationships present in our data. The clustering results using MDS on pseudoeigenfunctions were suboptimal, as MDS struggled to represent the true structure of the data. Consequently, the clusters identified by MDS did not align well with the underlying spectral characteristics.

The integration of this clustering method into the broader framework of EDMD and ResDMD provides a thorough and robust approach to the analysis of complex dynamical systems. It is specifically designed for systems characterized by continuous spectra, providing a refined perspective on their dynamics. The design of the method ensures that it is accessible and understandable to a wide audience, including both domain experts and individuals with a general interest in dynamical systems.

### Supplementary Information

Supplementary Information.

## Data Availability

All data generated and analyzed during this study are included in this published article and its supplementary information files.
